# Transmembrane Protein 100 Expression on Endothelial Cells Vascularizing Thrombi in Chronic Thromboembolic Pulmonary Hypertension Modulates TGFβ1−ALK1 Signaling During Angiogenesis

**DOI:** 10.1002/pul2.70253

**Published:** 2026-01-28

**Authors:** Magdalena L. Bochenek, Iman Ghasemi, Christoph B. Wiedenroth, Olympia Bikou, Ioannis Karampinis, Eric D. Roessner, Lukas Hobohm, Stefan Guth, Philipp Lurz, Stavros Konstantinides, Katrin Schäfer

**Affiliations:** ^1^ Department of Cardiology, Cardiology I University Medical Center Mainz Mainz Germany; ^2^ Center for Thrombosis and Hemostasis, University Medical Center Mainz Mainz Germany; ^3^ German Center for Cardiovascular Research, Partner Site RhineMain Mainz Germany; ^4^ Department of Thoracic Surgery Kerckhoff Heart and Thorax Center Bad Nauheim Germany; ^5^ Department of Medicine I LMU University Hospital Munich Germany; ^6^ German Center for Cardiovascular Research, Partner Site Munich Munich Germany; ^7^ Department of Thoracic Surgery Center for Thoracic Diseases, University Medical Center Mainz Mainz Germany

**Keywords:** ALK1, chronic thromboembolic pulmonary hypertension, endothelium, TGF‐beta, TMEM100

## Abstract

Endothelial cells within chronic pulmonary artery thrombi in CTEPH overexpress transmembrane protein 100 (TMEM100), an activin A receptor‐like kinase 1 (ACVRL1 or ALK1) signaling‐dependent gene, and TGFβ1 upregulated TMEM100 transcription in healthy lung ECs. TMEM100 permitted the TGFβ1‐induced increase of ALK1, while repressing ALK5, and preventing ALK1–TMEM100 signaling impaired angiogenesis ex vivo. Our data indicate that TGFβ1–ALK1–TMEM100 signaling is active during CTEPH thrombus revascularization.

**Abbreviations**
ALK1: activin A receptor‐like type 1
ALK5: activin A receptor‐like type 5
CTEPH: chronic thromboembolic pulmonary hypertension
CTEPH‐ECs: CTEPH endothelial cells
EC: endothelial cells
HPAECs: human pulmonary arterial endothelial cells
HSaVECs: human saphenous vein endothelial cells
PA: pulmonary artery
PE: pulmonary embolism
PEA: pulmonary endarterectomy
TGFβ: transforming growth factor beta
TMEM100: transmembrane protein 100

## 1 Introduction

**Figure 1:**
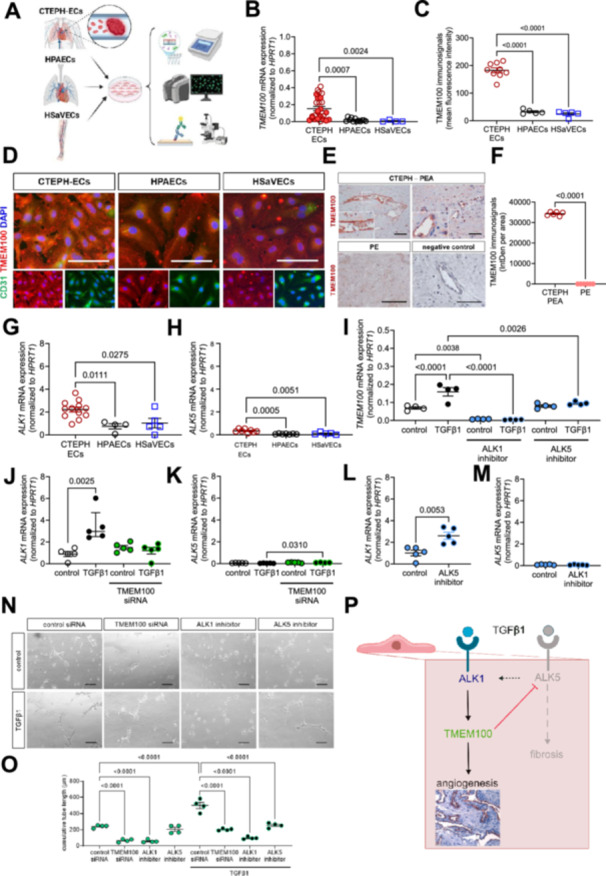
(A) Schematic drawing depicting the analysis of CTEPH‐ECs, HPAECs, and HSaVECs. The panel was created with BioRender.com. (B) *TMEM100* mRNA expression in CTEPH‐ECs (*n* = 20 biological replicates; ECs isolated from central PEA samples marked with “full” and distal PEA samples with “open” circles) compared to HPAECs (*n* = 3 biological replicates examined in 3–6 independent experiments) and HSaVECs (*n* = 1 biological replicate examined in four independent experiments). (C and D) TMEM100 protein expression in CTEPH‐ECs (*n* = 3 biological replicates examined in 3 independent experiments), HPAECs (*n* = 2 biological replicates examined in 2*–*3 independent experiments), and HSaVECs (*n* = 1 biological replicate examined in 5 independent experiments). Quantitative analysis (C). Representative images (D). Expression of CD31/PECAM1 is shown to demonstrate EC marker expression. (E and F) Immunohistochemical detection of TMEM100 on cross‐sections through PEA specimens from patients with CTEPH (*n* = 6 biological replicates) or PE (*n* = 3 biological replicates examined in 2 independent experiments). Representative images (E). Quantitative analysis (F). Findings after omission of the first antibody (negative control) are also shown. *ALK1* (G) and *ALK5* (H) mRNA expression in CTEPH‐ECs (*n* = 12 and *n* = 8 biological replicates, respectively) versus HPAECs (*n* = 3 biological replicates examined in 2–3 independent experiments) and HSaVECs (*n* = 1 biological replicate examined in 5 independent experiments). (I) *TMEM100* mRNA expression in HPAECs (*n* = 2 biological replicates examined in 2 independent experiments), control or after TGFβ1 stimulation (10 ng/mL for 24 h), alone or in the presence of the ALK1 inhibitor K02288 (10 µM) or the ALK5 inhibitor SB431542 (10 µM). *ALK1* (J) and *ALK5* (K) mRNA expression in HPAECs (*n* = 2 biological replicates examined in 3 independent experiments), control or after TGFβ1 stimulation, and TMEM100 siRNA transfection. *ALK1* (L) and *ALK5* (M) mRNA expression in HPAECs (*n* = 2 biological replicates examined in 2–3 independent experiments), alone or in the presence of an ALK5 (L) or ALK1 (M) inhibitor. Representative images (N) and quantitative analysis of the cumulative tube length (O) of CTEPH‐ECs (*n* = 1 biological replicate examined in 4 independent experiments) cultivated on Matrigel for 5 h and the effects of TGFβ1 stimulation (10 ng/mL for 24 h), control or TMEM100 siRNA transfection, ALK1, or ALK5 inhibition. Exact *p* values were determined using unpaired Student's *t*‐test (F, L, and M), one‐way ANOVA, Sidak's multiple comparisons test (G, H, I, and O), or Kruskal–Wallis, Dunn's multiple comparisons test (B, C, J, and K), based on the Shapiro–Wilk test for normal distribution. Quantitative data are shown as mean ± SEM or median with interquartile range, depending on the presence of normal distribution or not. Scale bars in (D), (E), and (N) denote 100 µm. (P) Visual summary of the findings of the study. The panel was created with Inkscape v.1.4.2.

Pulmonary embolism (PE) is a life‐threatening, acute cardiovascular event, caused by the occlusion of parts of the pulmonary arterial (PA) tree with thrombus material originating from the deep veins or the right heart. Chronic thromboembolic pulmonary hypertension (CTEPH) develops as a late complication of PE [1, 2]. Histologically, CTEPH is characterized by fibrotic scar tissue interspersed with thrombi of varying age and resolution state, infiltrating immune cells, and enlarged and irregularly shaped vascular structures [3]. These so‐called angioproliferative lesions can be observed in CTEPH and other forms of PH [4] and reflect an abnormal growth of blood vessels. Work by our group and others has highlighted the importance of endothelial cells (ECs) for venous thrombus revascularization and resolution [5], including the activation of endothelial signaling pathways downstream of transforming growth factor (TGF)‐β [6]. However, the cellular and molecular mechanisms that prevent complete thrombus resolution in the pulmonary vasculature remain poorly defined [7], owing also to the complexity of TGFβ receptors, ligands and downstream effectors and cell type‐dependent specificities [8]. Recent profiling of the organ‐specific transcriptome on the single cell level uncovered transmembrane protein 100 (TMEM100), an activin A receptor‐like kinase 1 (ACVRL1 or ALK1) signaling‐dependent gene [9], as one of the genes highly enriched on ECs of the murine lung [10, 11]. Lineage tracing in mice confirmed TMEM100 as lung‐specific endothelium gene [12]. Here, we reasoned that analyzing the expression, localization and regulation of TMEM100 in human lung and non‐lung ECs as well as EC from pulmonary endarterectomy (PEA) specimens of patients with CTEPH (CTEPH‐ECs; Figure 1A) will allow insights into their origin, but also give hints for potential pathomechanisms related to endothelial ALK1 signaling and its importance for pulmonary thrombus vascularization and organization.

## 2 Methods

CTEPH‐ECs (Passage 1; Ethics Approval AZ 199/15) were isolated from CTEPH PEA specimens, as described in Bochenek et al. [13], and compared to human pulmonary arterial ECs (HPAECs; PromoCell, C‐12241, Lot Numbers 458Z016.13 and 482Z007.13; ATCC, PCS‐100‐022, Lot Number 59880292) and ECs from the human saphenous vein (HSaVECs; PromoCell, C‐12231, Lot Number 455Z015.1), a frequent origin of blood clots embolizing into the lungs. ECs cultured on glass coverslips precoated with 0.2% gelatin were fixed and stained using monoclonal antibodies against TMEM100 (clone OTI10G3; RRID: AB_2725530; ThermoFisher Scientific; MA5‐24949; dilution, 1:100) and CD31/PECAM1 (clone MEC13.3; SantaCruz Biotechology, sc‐18916; dilution, 1:50). Paraffin‐embedded specimens from CTEPH patients or thrombotic material percutaneously removed via embolectomy from patients with acute PE were deparaffinized and stained using TMEM100 antibody (dilution, 1:100). All patients, who underwent PEA or embolectomy gave written informed consent (Ethics Approval AZ 199/15, Amendment November 14, 2023, and AZ 2023‐17125, September 15, 2023). Immunohistochemical (IHC) images were collected using an Olympus BX51 light microscope, and immunofluorescent images using a high‐resolution Keyence BZ‐X800 fluorescence microscope. Images were analyzed using ImageJ software, and the results are shown as integrated density (IntDen for IHC) or mean fluorescence intensity (MFI for IF), which is calculated by the measurement of total fluorescence divided by number of cells in the image. ECs in culture were lysed using Trizol reagent (Ambion). RNA was isolated and reverse transcribed into cDNA followed by quantitative real‐time polymerase chain reaction and SYBR Green (Bio‐Rad Laboratories) to examine the mRNA expression of *ALK1*, *ALK5*, and *TMEM100*. All qPCR data were normalized to the housekeeping gene *HPRT1* and are reported as relative expression levels. In some experiments, cells were preincubated with the ALK1 inhibitor K02288 (10 µM; Selleckchem; #S7359) or the ALK5 inhibitor SB431542 (10 µM; Tocris; #1614). The ability of ECs to proliferate, migrate and to form tube‐like structures and networks was examined using the Matrigel angiogenesis assay. To determine the importance of TMEM100, HPAECs were transfected with TMEM100 siRNA (ID#140542; ThermoFisher Scientific; AM16708) or with Silencer Negative Control #1 (ThermoFisher Scientific; AM4611) using Lipofectamine RNAiMax transfection reagent (ThermoFisher Scientific, #13778030). Forty‐eight hours after transfection, cells were trypsinized with 0.25% Trypsin–EDTA (Gibco; #25200056) and plated onto Matrigel (Corning; #356237) in 24‐well plates (at a density of 1 × 10^4^ cells/well). Five hours postplating, images were taken, and the total number of nodes and total network length per microscope as a measure of network complexity was quantified using ImageJ software. Results are shown as mean ± standard error of the mean (SEM), if normally distributed, or as median with interquartile range, if not. Normality and Lognormality were determined based on the Shapiro–Wilk test for normal distribution. If two groups were compared, Student's *t*‐test was used for normally distributed values (Figure 1F,L,M). If more than two groups were compared, one‐way ANOVA followed by Sidak's multiple comparisons test (Figure 1G,H,I,O) was used for normally distributed values, and Kruskal–Wallis followed by Dunn's multiple comparisons test for non‐normally distributed values (Figure 1B,C,J,K). The data were analyzed with GraphPad Prism, Version 10.4.1.

## 3 Results

Quantitative real‐time PCR (Figure 1B) and immunofluorescence microscopy (Figure 1C,D) showed significantly upregulated *TMEM100* mRNA and TMEM100 protein levels in CTEPH‐ECs compared to HPAECs and HSaVECs. ECs lining the enlarged, irregular vascular structures within the PEA material removed from the pulmonary arteries of patients with CTEPH also strongly expressed TMEM100, whereas vascular structures were not (yet) present in the thrombotic material removed from patients with acute PE (Figure 1E,F). These findings, together with the earlier characterization of lung endothelium‐specific expression patterns of TMEM100 [10, 11, 12], suggest that CTEPH‐ECs revascularizing the chronic thrombi in CTEPH originate from the lung. A recent study showed that subsets of vessels recanalizing subsegmental pulmonary arterial lesions in CTEPH express the systemic endothelial marker collagen 15A1 as well as COL15A1 negative cells, suggesting that “ECs from both the pulmonary and the systemic circulation contribute to lesion recanalization” [14]. Of note, *TMEM100* mRNA levels did not significantly differ between CTEPH‐ECs isolated from distal or central PA compartments (data in Figure 1B).

Work from others has shown that TMEM100 is essential for normal blood vessel development downstream of ALK1 [9]. We have shown that activated TGFβ signaling via ALK1 and ALK5 promotes venous thrombus nonresolution and the formation of fibrotic, vessel‐rich thrombi resembling those in CTEPH [6]. Here, we show that mRNA levels of *ALK1* (Figure 1G) and *ALK5* (Figure 1H) are significantly upregulated in CTEPH‐ECs compared to HPAECs and HSaVECs. Of note, mean *ALK1* mRNA transcript levels were approximately ten‐fold higher than those of *ALK5*. ALK1 and ALK5 are both type I receptors in the TGFβ signaling pathway, but ALK1 is highly expressed on ECs, whereas ALK5 expression is observed on a wider range of cell types [15].

Stimulation of HPAECs with TGFβ1 (10 ng/mL for 24 h) significantly increased *TMEM100* (Figure 1I) and *ALK1* (Figure 1J) mRNA transcript levels, whereas ALK5 mRNA levels did not change in response to TGFβ1 (Figure 1K), strongly suggesting a role for TGFβ1 as upstream regulator of the ALK1–TMEM100 signaling axis and that the overexpression of TMEM100 and ALK1 in CTEPH‐ECs occurs downstream of TGFβ1. Inhibition of ALK1 or ALK5 both prevented the increase of TMEM100 expression in response to TGFβ1; however, only ALK1 inhibition resulted in lower *TMEM100* mRNA levels already under steady‐state conditions, whereas inhibition of ALK5 did not play a role (Figure 1I). As expected, loss of TMEM100 (achieved by siRNA) abolished the TGFβ1‐induced increase in *ALK1* mRNA expression (Figure 1J), whereas *ALK5* mRNA levels significantly increased in response to TGFβ1 stimulation only in the absence of TMEM100 (Figure 1K). The latter finding suggests that TMEM100 acts as a signaling node to promote TGFβ1 signaling via ALK1 while suppressing signaling via ALK5. qPCR findings that inhibiting ALK5 significantly upregulated *ALK1* levels (Figure 1L) support this conclusion and also suggest that the comparatively low *ALK5* expression on CTEPH‐ECs (data in Figure 1H vs. Figure 1G) diverts TGFβ1 signaling toward the ALK1–TMEM100 pathway. On the other hand, ALK1 inhibition did not induce *ALK5* expression (Figure 1M), in line with earlier studies showing that ALK5 is not required to produce a vascular phenotype [16]. Baseline *ALK1* and *ALK5* mRNA expression was not changed by the absence of TMEM100.

To evaluate the importance of the TGFβ1–ALK1–TMEM100 axis for endothelial functions during thrombus revascularization, we employed the Matrigel assay to study angiogenic network formation by CTEPH‐ECs ex vivo. The cumulative network length and number of nodes (not shown) significantly increased in response to TGFβ1, and both were prevented by TMEM100 siRNA transfection or ALK1 inhibition, whereas ALK5 inhibition had no effects in control‐treated cells, but prevented the angiogenic response to TGFβ1 (Figure 1N,O).

## 4 Discussion

In summary, we show that ECs vascularizing the obstructive thrombotic material in the pulmonary artery of patients with CTEPH express high levels of the lung endothelial‐specific marker TMEM100, downstream of endothelial TGFβ1 signaling via ALK1, and that TGFβ1–ALK1–TMEM100 signaling enables the sprouting and angiogenic network formation of CTEPH‐ECs ex vivo. On the other hand, our data also suggest that TMEM100 actively represses ALK5, supporting the concept that the imbalance between ALK1 and ALK5 signaling and thus, the activation and resolution phase of angiogenesis [17], determines the revascularization of chronic pulmonary thrombi in CTEPH (Figure 1P). Our findings are in line with the reported opposing roles of ALK1 and ALK5 in regulating endothelial functions [15] and their reciprocal interaction [18]. TGFβ1 signaling via ALK1 promotes endothelial proliferation [19], whereas signaling via ALK5 was shown in pulmonary microvascular ECs to cause apoptosis [20]. The accessory TGFβ receptor protein endoglin was shown to mediate the repression of endothelial ALK5 signaling [19]. Endothelial TGFβ1 signaling via ALK5 also activates the transcription of endothelin‐1 [21] and promotes venous thrombofibrosis [13], and thus contributes to the pathophysiology of CTEPH via mechanisms not involving TMEM100. Previous work has shown that TMEM100 is an ALK1‐dependent gene [9], but this is the first report that the ALK1–TMEM100 axis is functionally active in CTEPH‐ECs. We show that TMEM100 is highly expressed in CTEPH‐ECs and ECs lining the enlarged vascular structures within the obstructive pulmonary thrombi in situ, and demonstrate its relevance for angiogenesis using loss‐of‐function (siRNA, inhibitors) approaches or TGFβ1 stimulation as an upstream regulator to increase endothelial TMEM100 expression. Our study also has some limitations: HPAECs were obtained from healthy human donors via commercial providers and not from an unaffected area within the pulmonary artery of the same patient. Although it may be considered an ideal internal control, this was not possible due to ethical restrictions. Also, our analyses were performed ex vivo and thrombus vascularization, gene expression and the response to stimuli may differ under in vivo conditions. Taken together, the results of our analyses not only advance our understanding of the pathophysiology of CTEPH, but also may help to refine its treatment by targeting endothelial signaling in thrombus resolution.

## Author Contributions

**Katrin Schäfer:** conceptualization, writing – original draft preparation, writing – review and editing, supervision, project administration, funding acquisition. **Magdalena Bochenek:** conceptualization, investigation, writing – original draft preparation, writing – review and editing, supervision, project administration, methodology, formal analysis. **Iman Ghasemi:** formal analysis, investigation, writing – review and editing. **Christoph B. Wiedenroth:** investigation, writing – review and editing, funding acquisition. **Olympia Bikou:** investigation, writing – review and editing. **Ioannis Karampinis:** investigation, writing – review and editing. **Lukas Hobohm:** investigation, writing – review and editing. **Eric Roessner:** resources, writing – review and editing. **Stefan Guth:** resources, writing – review and editing. **Philipp Lurz:** resources, writing – review and editing. **Stavros Konstantinides:** resources, writing – review and editing, funding acquisition.

## Ethics Statement

The study protocol was approved by the Institutional Ethics Committees (AZ 199/15, amendment November 14, 2023, and AZ 2023‐17125, September 15, 2023).

## Conflicts of Interest

C.B. Wiedenroth has received speaker fees or consultant honoraria from AOP‐Health, Bayer, Inari, J&J, MSD, OrphaCare, and Pfizer.
